# A comparative analysis of pretrained Wav2Vec XLSR-53 and Whisper-Small models for automatic speech recognition in the Telugu language

**DOI:** 10.3389/frai.2026.1878977

**Published:** 2026-07-30

**Authors:** Jagalingam Pushparaj, Muzaffar Ahmad Dar, Sri Gani Kaarthikeya Kammula, Anvita Manne, Arpit Tiwari, Gunasekhar Devineni, Chiranjeevi Karanki

**Affiliations:** 1 Department of Quantum AI, School of Computer Science and Engineering, Vellore Institute of Technology, Vellore, Tamil Nadu, India; 2 Department of Mathematics, School of Advanced Sciences, Vellore Institute of Technology, Vellore, Tamil Nadu, India

**Keywords:** automatic speech recognition, fine-tuning, Telugu language, Wav2Vec XLSR-53, Whisper-Small

## Abstract

This study presents the development of an automatic speech recognition (ASR) system tailored for Telugu, one of the widely spoken Indian languages. In recent years, deep learning (DL) techniques have been applied to develop ASR systems across various languages and domains. These models, however, require substantial training resources and extensive corpora of continuous speech composed from multiple dialectal speakers, along with their corresponding transcripts. This paper investigates the effectiveness of pre-trained models like Wav2Vec XLSR-53 and Whisper-Small for developing ASR systems for the Telugu language, addressing the challenge of limited data availability and demonstrating satisfactory results even when fine-tuned on a smaller dataset. We utilized approximately 20 h of speech data comprising 17,421 sentences of the Telugu language. The models are fine-tuned on four publicly available datasets, including OpenSLR, Common Voice, IndicVoices, and IndicTTS, to introduce greater diversity in both speaker demographics and linguistic content. The Wav2Vec XLSR-53 model achieved a word error rate (WER) of 27.3% and a character error rate (CER) of 6.8% on the test dataset, whereas the Whisper-Small attained a WER of 28.67% and a CER of 7.55%. In addition, performance of the models was evaluated by introducing noise to both individual datasets as well as a combined noise dataset. The results show that, on the combined noise dataset, Wav2Vec XLSR-53 achieved a WER of 19.59% and a CER of 4.58%, while Whisper Small obtained a lower WER of 13.97% and a CER of 3.54%. These results underscore the usefulness of leveraging pre-trained architectures in low-resource linguistic scenarios such as Telugu.

## 1 Introduction

ASR is a machinery that changes vocal language into printed text, enabling applications such as voice assistants (e.g., Siri, Alexa) and real-time transcription services. Modern ASR systems, including OpenAI's Whisper (Radford et al., 2022), utilize DL models trained on large-scale audio-text datasets to achieve high accuracy in speech recognition. Since its inception in the 1950s, ASR has evolved from rule-based approaches to sophisticated neural architectures capable of handling diverse accents and languages. ASR systems continue to face trials such as backdrop noise, speaker inconsistency, and complex linguistic structures, which contribute to transcription errors (Hinton et al., 2012). A significant advancement in recent years is self-supervised learning (SSL), exemplified by models like Wav2Vec 2.0 (Conneau et al., 2020), which extract meaningful representations from raw audio, reducing reliance on extensive labeled datasets.

Developing accurate ASR systems remains challenging due to factors such as environmental noise, speaker diversity, linguistic complexity, and accent (Ahmad Dar and Pushparaj, 2025). Noisy environments (e.g., cafes, traffic) introduce overlapping sounds that distort speech signals, making it difficult for models to segment and recognize words (Vincent et al., 2006). Speaker variability further complicates ASR performance–systems trained on limited datasets often struggle with dialects and code-switching (seamless transitions between languages within a sentence) (Ardila et al., 2020). Ethical concerns also arise, as biased training data can affect non-native speakers and underrepresented linguistic communities. Also, low-resource languages, including many regional tongues, suffer from a lack of labeled audio data, limiting the development of robust ASR models. Even for widely spoken languages, computational constraints and the trade-off between real-time processing and transcription accuracy remain significant challenges.

Recent advancements in sequence-based models have transformed ASR by improving efficiency and accuracy. Unlike traditional systems that rely on multiple intermediate steps, modern neural network architectures, such as the transformer framework (Vaswani et al., 2017), map raw audio to text, streamlining the recognition pipeline. These frameworks emulate human speech processing by capturing contextual relationships between phonetic units, enhancing transcription accuracy for high-resource languages like English. To address data scarcity, researchers have adopted SSL to enable ASR models to learn from enormous volumes of unlabeled speech data, revolutionizing recognition performance for under-resourced languages (Dar and Pushparaj, 2025). Instead of depending on manually transcribed data, SSL models uncover latent patterns in raw audio, making them adaptable to languages with unique phonetic structures. However, fine-tuning these models on the Telugu language still necessitates dialect-specific adaptations, such as customizing language models to accommodate regional vocabulary, complex script, dialectal diversity (e.g., Coastal vs. Telangana accents), and limited digital resources (Satla and Manchala, 2021).

Hybrid approaches, including deep neural network-hidden Markov model (DNN-HMM) acoustic modeling, continue to refine large-vocabulary ASR systems, ensuring that Telugu speech recognition keeps pace with global advancements. Compared to English, Telugu presents unique challenges for ASR. English employs a simple Latin alphabet with 26 letters and straightforward phoneme-to-text mappings, while the Telugu language, spoken by over 80 million people, utilizes a syllabic script with 56 base characters and intricate conjunct consonants (e.g., “kka” or “thha”) formed through character combinations (Toshniwal et al., 2018). Also, Telugu's use of matras (vowel modifiers) alters pronunciation dynamically (e.g., “ki” vs. “kee”), requiring ASR models to accommodate a more extensive phonetic inventory and complex grammatical structures.

Integrating advanced techniques into low-resource ASR systems presents both opportunities and challenges. Cross-lingual knowledge transfer in DL offers promising solutions for improving Telugu ASR by leveraging high-resource language models. However, the scarcity of labeled datasets remains a major bottleneck, limiting the capacity to adjust pre-trained architectures for Telugu. In this study, we focus on fine-tuning Wav2Vec XLSR-53 and Whisper-Small to enhance their capacity for Telugu speech recognition. By harnessing their advanced feature extraction capabilities, we aim to overcome the unique linguistic challenges associated with Telugu, thereby improving ASR performance in low-resource settings.

The contributions of the presented work are

- We curated data from four publicly available sources: OpenSLR, Common Voice, IndicVoices, and IndicTTS. The combined data increases diversity in speaker demographics and linguistic content that covers all Telugu language dialects.
- We performed an extensive ablation study over the hyperparameters like learning rate, batch size, and optimizer, detailing how each factor impacts WER and CER for the models, offering hyperparameter recommendations for low-resource languages.
- We evaluated the robustness of the Wav2Vec XLSR-53 and Whisper-Small models under clean testing data and by adding background noise from the ESC-50 dataset to all four data source.
- We performed qualitative error analysis grounded in Telugu linguistics related to compound word handling, phonetic variations, and contextual nuances.

The remainder of this paper is structured as follows: Section 2 provides a review of related work. Section 3 describes the methodology, including data preparation, model architecture, and evaluation metrics. Section 4 provides the ablation study results and training configuration. Section 5 presents and discusses the experimental results as well as the model observation. Finally, Section 6 gives the conclusion of the study and suggests directions for future research.

## 2 Related studies

Recent ASR research has introduced diverse architectural and contextual improvements. While hybrid DNN-HMM systems are efficient, they often underperform compared to attention-based encoder-decoder (AED) models in terms of accuracy (Li et al., 2023). Security analyses have also revealed vulnerabilities in speech emotion recognition, with current safeguards mitigating only 30% of adversarial threats (Gurowiec and Nissim, 2024). Meanwhile, multi-sensory approaches such as vibrotactile feedback (Răutu et al., 2023) and lightweight online audio-visual frameworks (Sterpu and Harte, 2022) have enhanced noise robustness and computational efficiency, achieving practical trade-offs for real-world deployment. Also, innovations such as time-delay networks for voice classification (Kadiri et al., 2024) and adaptations of DeepSpeech for low-resource languages (24.7% WER) (Changrampadi et al., 2022), demonstrate ASR's expanding scope while balancing performance with computational constraints. The study (Duarte and Colcher, 2024) compared preprocessing techniques across signal, feature, and model domains to address the mismatch between training and testing data. The authors Abdel-Hamid et al. (2014) demonstrated that convolutional neural networks (CNNs) further reduce error rates by 6-10% compared to conventional DNNs, while Du et al. (2022) utilized domain adversarial training. Recent developments in self-supervised and transformer-based speech recognition paradigms have considerably enhanced the performance of low-resource ASR systems (Agoujil and Yousfi, 2025).

Developing ASR systems for low-resource languages remains a significant challenge, as these languages often lack extensive annotated datasets. The availability of superior speech corpora endures a foremost contest for developing ASR systems in low-resource languages. Several speech datasets have newly been presented to enable research on underrepresented languages, including the Moroccan Darija Voice Corpus (MDVC) (Boumehdi and Yousfi, 2024). Several studies have explored ASR for various low-resource Indian languages, employing diverse DL architectures. In Paul et al. (2024), a bidirectional long short-term memory (BLSTM) model was applied to a Bangla language dataset, and the model achieved a recognition accuracy of 94.2%. Similarly, HindiSpeech-Net (Sharma et al., 2022), a seven-layer one-dimensional CNN, was designed for Hindi ASR using 2,400 samples distributed across 10 categories, achieving a test accuracy of 92.92%. Another study (Shivakumar et al., 2016) investigated ASR for the Kannada language and developed a baseline ASR system on a corpus of 1,000 annotated sentences by incorporating probabilistic language models.

Recent research has advanced Telugu ASR through improved collection of a large-scale Telugu speech dataset (Mirishkar et al., 2021) via mobile and web platforms, and baseline ASR performance was evaluated under clean and noisy conditions. A unified end-to-end ASR system for three Telugu dialects—Telangana, Coastal Andhra, and Rayalaseema (Yadavalli et al., 2022b)—proposed a unified solution for dialect variability by building a multi-task end-to-end ASR framework that jointly performs speech transcription and dialect identification by improving performance for low-resource dialects, with more than 9.71% relative improvement in WER. Another study (Chowdary et al., 2024a) developed a multilingual ASR model using Whisper, reporting a 61.2% WER. The study Yadavalli et al. (2022a) focused on three major Telugu dialects: Telangana, Coastal Andhra, and Rayalaseema, and findings showed that both acoustic models and language models suffer under dialect-mismatched conditions. A comparative study by Chowdary et al. (2024b) evaluated Wav2Vec XLSR-53 and Whisper-Small models for Telugu and other Dravidian languages, and results showed that Wav2Vec XLSR-53 and Whisper-Small achieved relatively lower WER and CER.

Despite significant advancements in ASR, achieving robustness across diverse environments, languages, and computational constraints remains challenging. Resource limitations hinder Telugu ASR development–publicly available datasets remain scarce compared to those in English. Moreover, dialectal variations and frequent code-mixing with English introduce additional challenges. While SSL and hybrid adaptation techniques show promise, a unified framework is needed for improved ASR reliability. This paper focuses on developing an efficient ASR system for Telugu, leveraging the Wav2Vec XLSR-53 and Whisper-Small models trained on distinct datasets of the Telugu language.

## 3 Methodology

**Figure 1:**
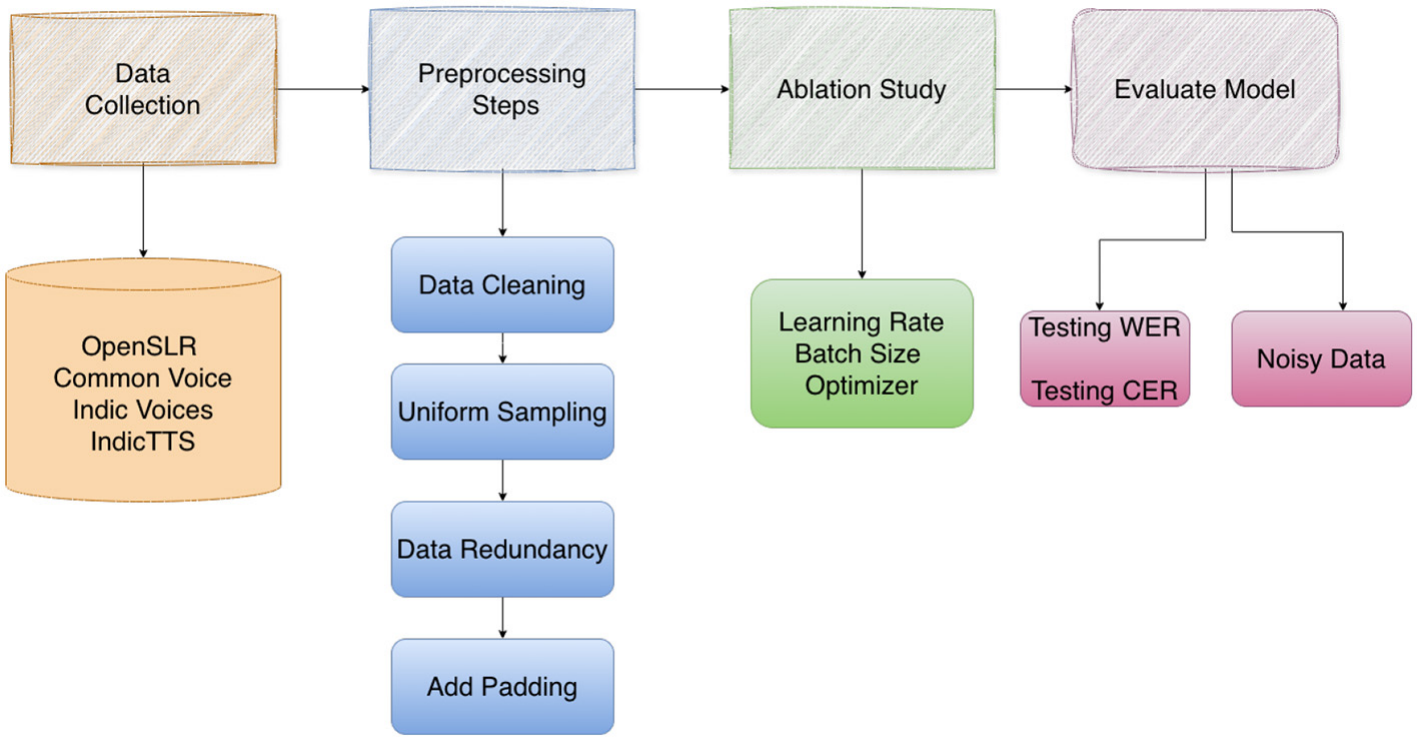
Overall methodology of the proposed work.

In this section, we provide an overview of the methodology employed in this study, which includes data collection, data preprocessing, a detailed description of the Wav2Vec XLSR-53 and Whisper-Small models, and the evaluation metrics used to assess their performance. The overall methodology of this work is illustrated in Figure 1.

### 3.1 Dataset description

**Figure 2:**
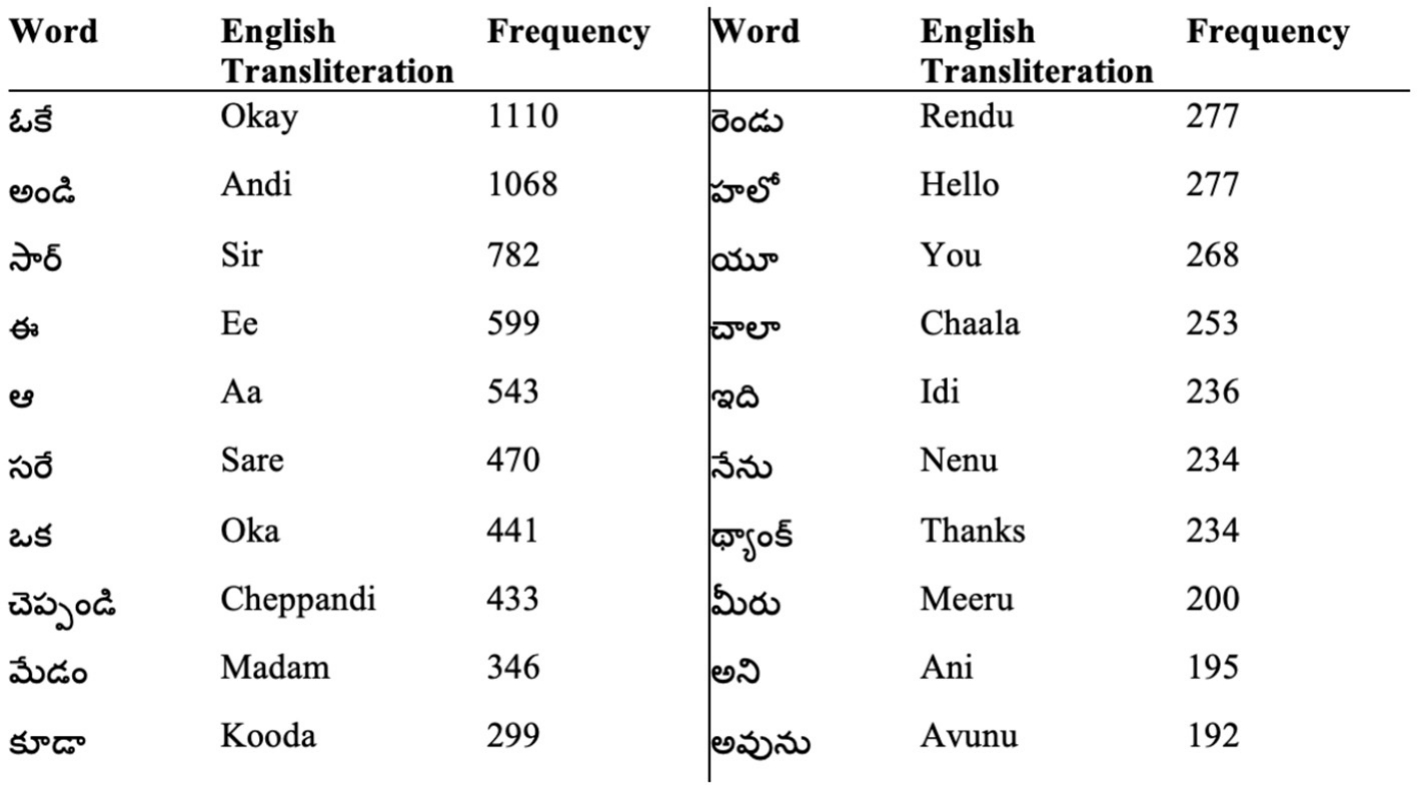
Top 20 most frequent words in the combined dataset.

Collecting data for low-resource languages such as Telugu poses considerable challenges due to the limited availability of labeled speech corpora. To address this constraint, we collected the data from the four publicly accessible datasets: OpenSLR (He et al., 2020), Common Voice (Ardila et al., 2019), IndicVoices (Javed et al., 2024), and IndicTTS (Kumar et al., 2023). The combined data from all four datasets introduced a greater diversity in both speaker demographics and linguistic content, and with this integration, reinforced the phonetic representation during model training. A comprehensive description of the datasets used for this study is provided in the corresponding Table 1. It includes detailed statistics for each dataset. This information offers valuable insight into the linguistic and demographic diversity of the model training and evaluation data. In total, the combined dataset comprises 20 hours of speech, consisting of 15,811 training sentences and 1,610 testing sentences. The combined dataset comprises 69,127 word tokens, including 23,293 unique words and 50 unique phonemes, with a speaker distribution of 122 males and 109 females. Figure 2 illustrates the 20 most frequently occurring words in the combined dataset, accompanied by their English transliterations and corresponding usage frequencies.

**Table 1:** Dataset details and description, including estimated dialectal distribution, of the data used for the development of the Telugu ASR.

Feature	OpenSLR	Common voice	IndicVoices	IndicTTS	Combined dataset
Train sentences	3,558	1,832	9,097	1,324	15,811
Test sentences	890	152	277	291	1,610
Total words	22,430	7,599	28,253	10,845	69,127
Unique words	7,935	1,347	8,593	5,418	23,293
Phonemes	45	38	50	37	50
No. of hours	6	4	7.5	2.5	20
Gender M-F	23-24	40-10	58-74	1-1	122-109
Dialect Dist. (%)	Unspecified	Unspecified	Coastal: ~35% Rayalaseema: ~25% Telangana: ~40%	~100% Coastal (Standard)	Coastal: ~26% Rayalaseema: ~9% Telangana: ~15% Unspecified: ~50%

Dialectal metadata is not provided for datasets like OpenSLR and Common Voice. The distribution for IndicVoices is estimated based on its goal of broad geographic coverage, as it is a corpus with speaker location metadata. The IndicTTS dataset is based on the Coastal dialect.

The Telugu language exhibits significant linguistic diversity, categorized into three major dialects: Coastal Andhra, Telangana, and Rayalaseema. Each dialect features unique variations in pronunciation and intonation, which can pose a considerable challenge for an ASR system. An ideal training corpus would contain a balanced representation of all major dialects to ensure robust performance across different speaker populations. In this study, we utilized datasets encompassing all dialects to ensure that the model learns from the full range of dialectal variations. Table 1 presents the information about the datasets used in this study.

### 3.2 Data preprocessing and unification

The four datasets used in this study were recorded under different conditions and exhibited inconsistencies in sampling rates and audio quality. To create a unified and consistent dataset for model training, a standardized preprocessing pipeline was applied to all audio files using the Python Librosa library. The following steps were performed:

- **Sampling rate adaptation:** all audio recordings were resampled to a unvarying sampling rate of 16 kHz, which is the standard input requirement for the models
- **Channel unification:** all stereo recordings were converted to a single (mono) channel.
- **Format and bit-depth standardization:** all files were converted and saved in the WAV format with a 16-bit pulse code modulation (PCM) encoding to ensure consistency across the entire dataset.
- **Transcript normalization:** the corresponding transcripts were cleaned by removing all punctuation, converting text to lowercase, and expanding any numerals or symbols into their full word equivalents to ensure a clean mapping between the audio and text.

For the Wav2Vec XLSR-53 model, preprocessing extended beyond standard normalization and cleaning. Transcripts were tokenized into phoneme-level units and padded to uniform lengths, enabling efficient sequence modeling. This process was carefully designed to preserve the linguistic characteristics of the Telugu language, ensuring that phonetic nuances were captured. In contrast, the Whisper-Small model adopted a more streamlined approach, leveraging raw audio inputs and their corresponding transcriptions without explicit tokenization or padding. To maintain data integrity, an additional validation step was incorporated, ensuring proper alignment between audio and text pairs while preventing transcription mismatches.

### 3.3 Dataset splitting and leakage prevention

To ensure the integrity of our evaluation and to obtain a reliable measure of the models' robustness, it is crucial to prevent any data leakage between the training and test sets. We implemented a speaker-disjoint splitting strategy.

- For OpenSLR, Common Voice, and IndicVoices: we performed an 80%/20% split based on speaker IDs, ensuring that all utterances from any individual speaker were placed in either the training or test set. This approach eliminates the risk of the model overfitting.
- For IndicTTS: since explicit speaker IDs were not available, we adopted a random split at the utterance level (80% training, 20% testing), and this approach is standard in such cases.

### 3.4 Wav2Vec XLSR-53 model description

**Figure 3:**
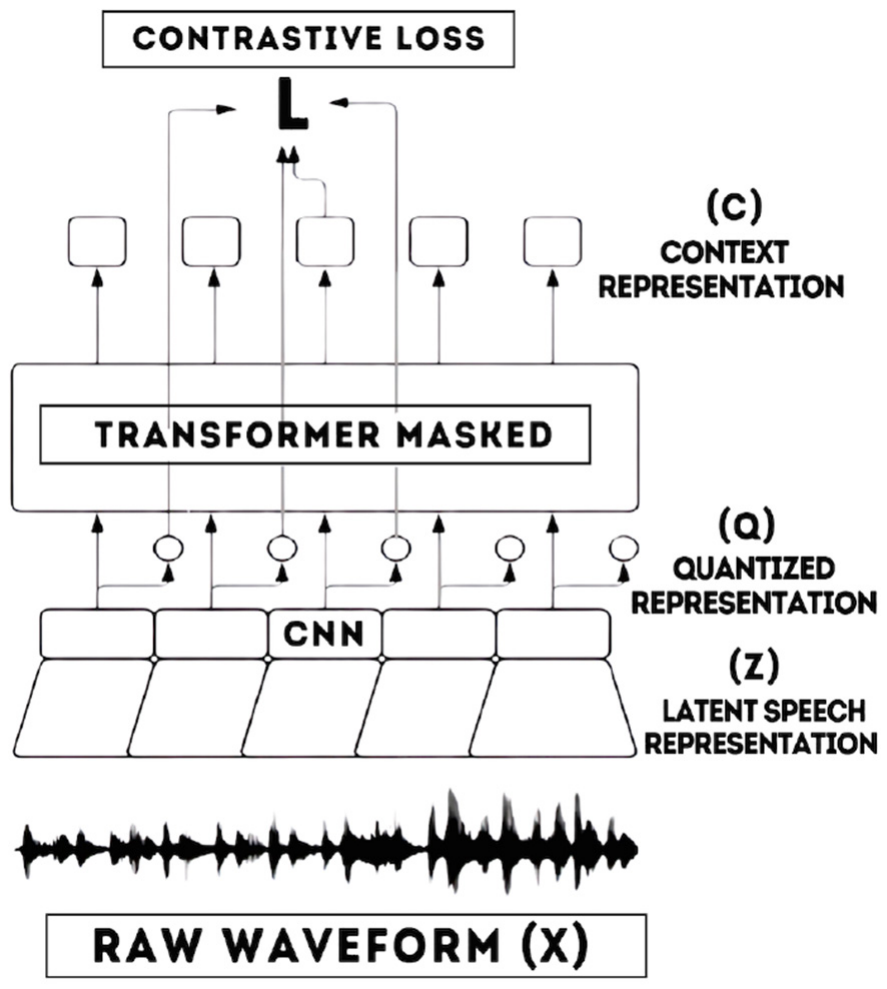
Wav2Vec XLSR-53 architecture: learning contextualized speech representations and discretized speech units (**?**).

Wav2Vec XLSR-53 (**?**) introduces a transformative approach to SSL in speech processing by leveraging large amounts of unlabeled audio data. It transforms raw audio into context-aware representations using a convolutional encoder, a quantization module, and a transformer network (see Figure 3). The SSL objective employs a contrastive loss to separate true representations from distractors. The framework comprises the following modules, as outlined below:

#### 3.4.1 Convolutional feature encoder

It transforms raw waveforms into latent speech representations by down-sampling the input audio *X* into latent features *Z*. This is accomplished through a series of convolutional layers, each optimized with specific strides and kernel sizes to capture temporal dependencies. The encoder extracts low-level acoustic features and outputs 512-dimensional latent representations, each corresponding to a 20 ms segment of audio.

#### 3.4.2 Quantization module

This module serves as an essential bridge between continuous latent representations and discrete embeddings, a crucial aspect of SSL. By employing the Gumbel-Softmax (Jang et al., 2016) technique, the model maps latent features *Z* to quantized variables *Q* while preserving differentiability. This approach allows the model to leverage discrete representations for training without disrupting gradient flow. The quantization process is mathematically defined as shown in Equation 1.


$$\begin{array}{c} q_{t}=\arg\max\left(\log\left(\pi_{i}\right)+g_{i}\right), \end{array}$$ (1)

Where *q*_*t*_ denotes the selected quantized vector, π_*i*_ represents the learned categorical distribution, and *g*_*i*_ is the Gumbel noise, which facilitates stochastic sampling.

#### 3.4.3 Transformer contextualizer

This module enhances the quantized latent representations by capturing global dependencies across the audio sequence. Utilizing self-attention mechanisms, it generates contextually enriched representations, which are beneficial for masked prediction tasks. Self-attention process is mathematically defined as in Equation 2:


$$\begin{array}{c} \mathrm{Attention}\left(Q,K,V\right)=\mathrm{softmax}\left(\frac{QK^{T}}{\sqrt{d_{k}}}\right)V, \end{array}$$ (2)

Where *Q, K, V* denote the query, key, and value matrices, respectively, and *d*_*k*_ represents the dimensionality of the key vectors.

#### 3.4.4 Masked prediction and contrastive loss

Wav2Vec XLSR-53 employs a masked prediction strategy analogous to BERT, where portions of the participation are covered, model is skilled to predict them. A contrastive loss function ensures that contextualized representations align with their true counterparts while remaining distinguishable from negative samples as shown in Equation 3.


$$\begin{array}{c} L_{c}=-\log\frac{\exp\left(\mathrm{sim}\left(c_{t},z_{t+1}\right)\right)}{\sum_{z_{k}\in Z_{\text{neg}}}\exp\left(\mathrm{sim}\left(c_{t},z_{k}\right)\right)}, \end{array}$$ (3)

Where sim(*c*_*t*_, *z*_*t*+1_) is the cosine similarity between *c*_*t*_ and *z*_*t*+1_, and *Z*_neg_ are the negative distractors.

#### 3.4.5 Fine-tuning for speech recognition

After pretraining, Wav2Vec XLSR-53 is fine-tuned for ASR by adding a linear classification layer that maps contextual representations to tokens. The model is optimized with the CTC Graves et al. (2006) loss as shown in Equation 4:


$$\begin{array}{c} L_{CTC}=-\log P\left(y|X\right), \end{array}$$ (4)

Where *P*(*y*|*X*), the probability of transcription is *y* given input *X*, yielding accurate downstream performance.

### 3.5 Whisper-Small model description

**Figure 4:**
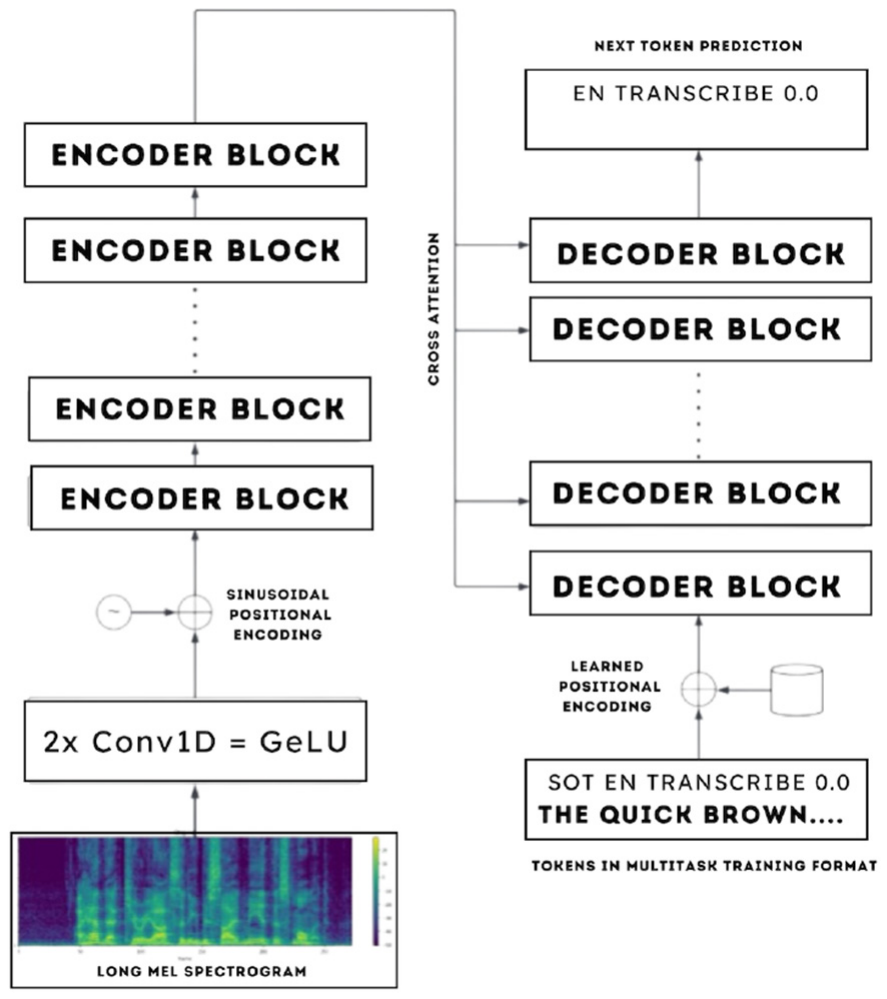
Whisper-Small encoder-decoder architecture (Radford et al., 2022).

The Whisper-Small (Radford et al., 2022) is built on an encoder-decoder, a standard transformer framework. Figure 4 shows the encoder-decoder architecture used for speech recognition. Input mel spectrograms are first processed by a 2D convolutional layer with GeLU activation and then augmented with sinusoidal positional encodings before entering the encoder stack. On the decoder side, learned positional encodings, cross-attention in each block, and a start-of-transcript token enable both transcription and related language tasks. The framework comprises the following phases, as outlined below:

#### 3.5.1 Encode phase

The encoder comprises 12 transformer blocks, each combining multi-head self-attention and a position-wise feed-forward network (FFN). Self-attention captures long-range dependencies across the mel sequence, computed as in Equation 2, while multi-head attention concatenates diverse head outputs. Each FFN applies two linear layers with a ReLU activation and residual connections, formulated as shown in Equations 5 and 6


$$\begin{array}{c} Z=\text{LayerNorm}\left(X+\text{MultiHead}\left(Q,K,V\right)\right) \end{array}$$ (5)

$$\begin{array}{c} \text{FFN}\left(Z\right)=\text{LayerNorm}\left(Z+\text{Linear}\left(\text{ReLU}\left(\text{Linear}\left(Z\right)\right)\right)\right) \end{array}$$ (6)

#### 3.5.2 Decoder phase

The decoder also uses 12 transformer blocks, adding cross-attention to align encoder outputs with previously generated tokens. Self-attention on the decoder input and cross-attention on encoder representations drive next-token prediction.

#### 3.5.3 Positional encoding

Sinusoidal positional encodings preserve sequence order in both encoder and decoder embeddings as shown in the Equations 7 and 8.


$$\begin{array}{c} PE\left(\text{pos},2i\right)=\sin\left(\frac{\text{pos}}{10000^{\frac{2i}{\text{model\_dim}}}}\right) \end{array}$$ (7)

$$\begin{array}{c} PE\left(\text{pos},2i+1\right)=\cos\left(\frac{\text{pos}}{10000^{\frac{2i}{\text{model\_dim}}}}\right) \end{array}$$ (8)

#### 3.5.4 Loss function

The model is optimized with cross-entropy loss (Deng and Woodland, 2024), which penalizes incorrect token predictions and promotes accurate transcription as shown in the Equation 9.


$$\begin{array}{c} L=-\frac{1}{N}\overset{N}{\underset{i=1}{\sum }}\log p\left(y_{i}|x\right), \end{array}$$ (9)

### 3.6 Evaluation metrics

To evaluate our fine-tuned ASR model, we use WER and CER. While WER is widely used for ASR evaluation, CER provides character-level insights (Mainzinger, 2024). WER is effective for English, but for agglutinative languages like Telugu, it may be less reliable. Thus, we report both metrics, noting their consistency across experiments. WER assesses word-level accuracy, while CER captures script-level fidelity. For morphologically complex languages, both are essential for optimizing ASR models.

#### 3.6.1 Word error rate (WER)

WER is a standard metric for quantifying the performance of ASR systems. It measures the word-level discrepancies between the predicted transcription and the ground truth by considering three types of errors: substitutions (*S*_*w*_), where an incorrect word replaces the correct one; insertions (*I*_*w*_), where extra words are erroneously added; and deletions (*D*_*w*_), where expected words are omitted. WER is calculated as shown in Equation 10.


$$\begin{array}{c} \text{WER}=\frac{S_{w}+I_{w}+D_{w}}{N_{w}} \end{array}$$ (10)

where *N*_*w*_ represents the total number of words in the reference transcript. A lower WER indicates better ASR performance.

#### 3.6.2 Character error rate (CER)

CER serves as an alternative evaluation metric that measures ASR performance at the character level. It evaluates transcription errors by comparing individual characters in the predicted output with those in the reference transcript. Like WER, CER considers substitutions (*S*_*c*_), where a character is incorrectly replaced; insertions (*I*_*c*_), where extraneous characters appear; and deletions (*D*_*c*_), where characters are missing. CER is computed using the formula as shown in the Equation 11:


$$\begin{array}{c} \text{CER}=\frac{S_{c}+I_{c}+D_{c}}{N_{c}} \end{array}$$ (11)

where *N*_*c*_ denotes the total number of characters in the reference transcript.

### 3.7 Computational costs

In this study, we evaluate the computational footprint of two ASR architectures, Wav2Vec XLSR-53 and Whisper-Small, for Telugu speech recognition. The Wav2Vec variant implemented here comprises roughly 300 million parameters, while Whisper-Small encompasses about 260 million. The experiments were conducted utilizing the RunPod platform, utilizing its cloud-based computational infrastructure. Both models were trained on Runpod's NVIDIA A100 GPU instances, each equipped with 40GB of HBM2e memory and high-bandwidth NVLink. Under these hardware conditions, Whisper-Small training required approximately 40 GPU-hours with peak memory usage near 30 GB, whereas Wav2Vec training utilized nearly half that, around 20 GPU-hours, peaking at about 35 GB.

## 4 Ablation study and training configuration

In the ablation study, key hyperparameters were explored to evaluate their impact on model performance. The hyperparameters considered included learning rate, batch size, and optimizer. To guarantee fair and reliable model comparison, all experiments, containing the ablation studies, applied early stopping and model check pointing. The validation loss was observed throughout training, and the checkpoint corresponding to the lowest validation loss was automatically saved. Training was completed when the validation loss unsuccessful to progress for the predefined epoch. All reported WER and CER values resemble to the finest validation checkpoint rather than the final training epoch. The configuration yielding the best performance was selected for the development of the final model.

### 4.1 Ablation study for Wav2Vec XLSR-53

**Figure 5:**
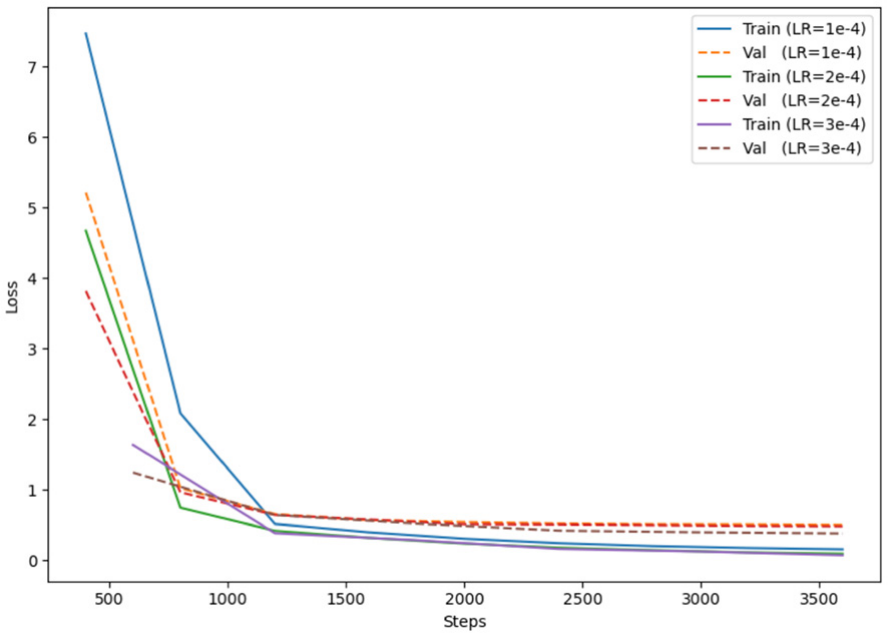
Training loss and validation Loss comparison between various learning rates for the Wav2Vec XLSR-53 model.

**Figure 6:**
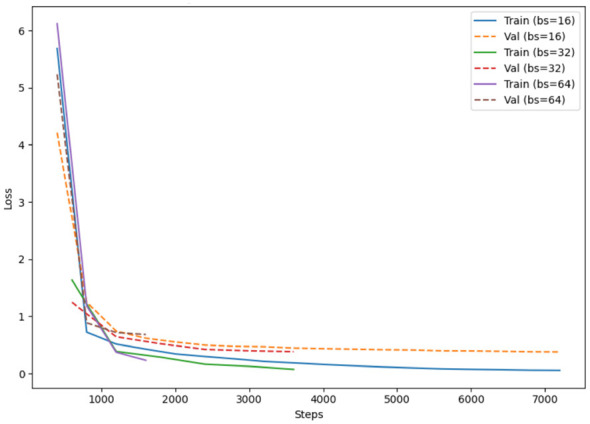
Training loss and validation loss comparison between various batch sizes for the Wav2Vec XLSR-53 model.

Tables 2–4 show the ablation study for Wav2Vec XLSR-53. This study experiments with choosing the hyperparameters for Telugu ASR. We found a learning rate of 3e-4 to be optimal–lower learning rates slowed convergence (WER jumped to 41.93% at 1e-4). A small batch size of 16 outperformed larger batches (WER 36.87% vs. 46.88% at size 64), and the RMSProp optimizer demonstrated better performance by achieving a WER of 27.3%, whereas the Adam optimizer reported a higher WER of 36.87%. The corresponding training and validation loss curves, shown in Figures 5, 6, confirm that the selected settings yield the fastest convergence with minimal overfitting. The optimal hyperparameter values are presented in bold in Tables 2–4.

**Table 2:** Ablation study for Wav2Vec XLSR-53 based on learning rate.

Learning Rate	WER	CER
**3e-4**	**38.13%**	**9.39%**
2e-4	38.77%	9.65%
1e-4	41.93%	10.25%

The optimal hyperparameter values are presented in bold.

**Table 3:** Ablation study for Wav2Vec XLSR-53 based on batch size.

Batch Size	WER	CER
**16**	**36.87%**	**9.17%**
32	38.13%	9.28%
64	46.88%	11.34%

The optimal hyperparameter values are presented in bold.

**Table 4:** An ablation study for Wav2Vec XLSR-53 based on optimizer.

Optimizer	WER	CER
Adam	36.87%	9.17%
**RMSProp**	**27.3%**	**6.8%**

The optimal hyperparameter values are presented in bold.

The observations of these ablation studies are as follows:

- **Learning rate:** a learning rate of 3e-4 performs satisfactorily with 38.13% WER and 9.39% CER when it is compared with (2e-4 and 1e-4). The higher learning rate provides the finest steadiness between convergence speed and stability, which helps the model to break the narrow minima while evading aggressive updating. On the other hand, the lower learning rates provide slightly higher WER and CER because it converge to lighter and sub-optimal minima.
- **Batch size:** the model achieves the finest WER of 36.87% and CER of 9.17% with a batch size of 16. This modest batch size delivers suitable steadiness with stochastic regularization and stability of the gradient. At the same time, when the batches were increased to 32 and 64, gradient noise was reduced, and the incline converged to sharper minima in the loss landscape, which can destructively disturb the performance of generalization performance.
- **Optimizers:** the RMSprop performs better because it adapts the learning rate based on current gradient statistics, which helps for the stability of training circumstances with elevated gradient variance. This stabilizes training when gradients fluctuate across time steps. Speech signals show high temporal variability, and Telugu adds phonetic complexity and duration variation; therefore, the RMSProp optimizer handles this variability better.

### 4.2 Ablation study for Whisper-Small

**Figure 7:**
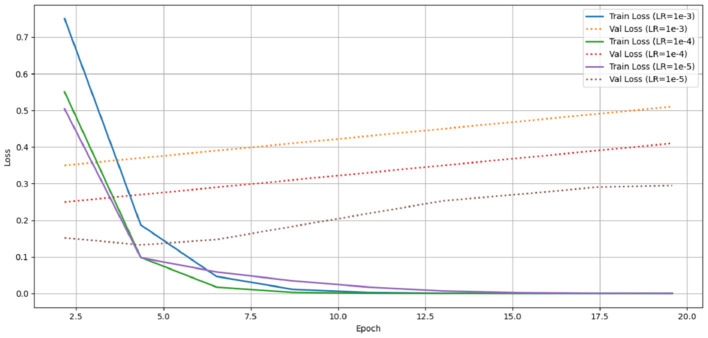
Training loss and validation loss comparison between various learning rates for the Whisper-Small model.

**Figure 8:**
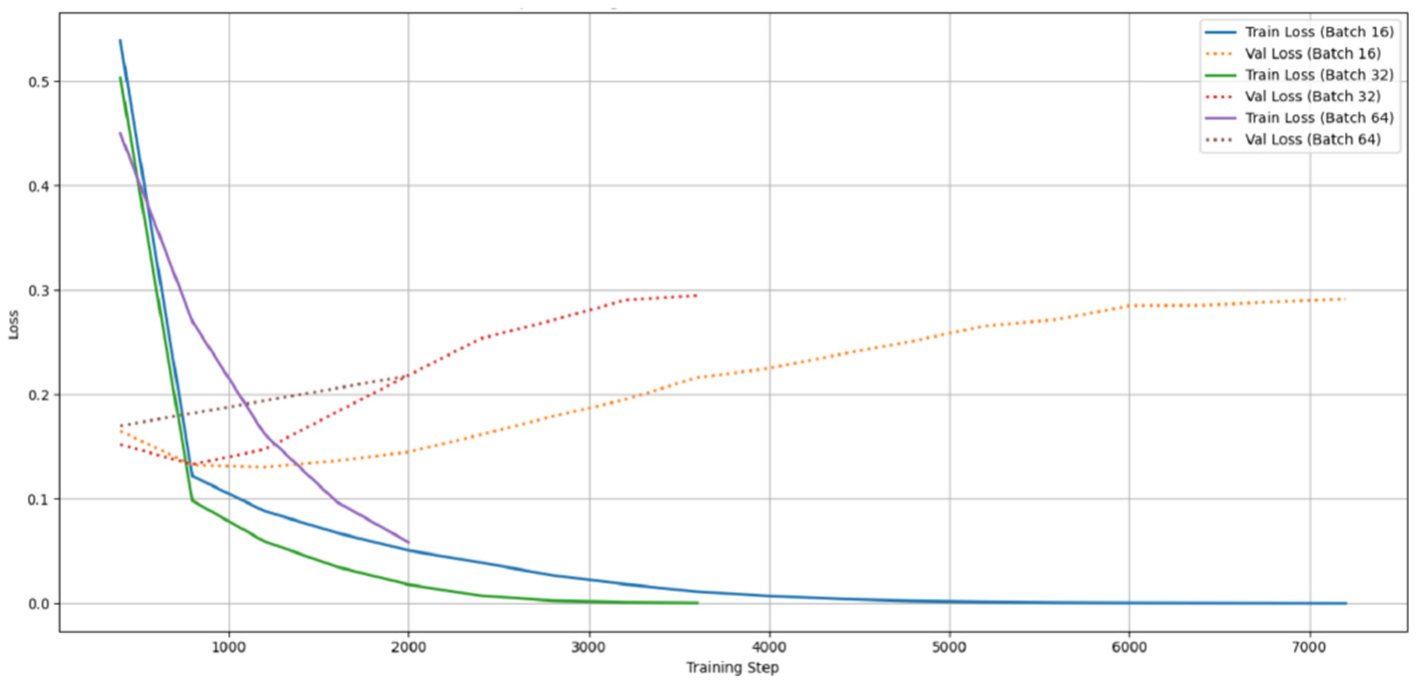
Training loss and validation loss comparison between various batch sizes for the Whisper-Small model.

Tables 5–7 show the ablation study for Whisper-Small. It tells a slightly different story and was most accurate at a very conservative learning rate (1e-5), with a WER of 40.87% and a CER of 10.37%; jumping the rate even modestly to 1e-4 shot error rates sky-high. Like Wav2Vec XLSR-53, a batch size of 16 and the RMSProp optimizer yielded the lowest errors before overfitting nudged performance back up. These loss curves–also shown in Figures 7, 8—show that Whisper-Small requires more cautious training schedules for its optimal spot, whereas Wav2Vec XLSR-53 tolerates more aggressive tuning without stability. In Tables 5–7, the optimal hyperparameter values are indicated in bold.

**Table 5:** Ablation study for Whisper-Small based on learning rates.

Learning Rate	WER	CER
**1e-5**	**40.87%**	**10.37%**
1e-4	51.67%	15.67%
1e-3	41.26%	10.86%

The optimal hyperparameter values are indicated in bold.

**Table 6:** Ablation study for Whisper-Small based on batch sizes.

Batch Size	WER	CER
**16**	**36.79%**	**9.92%**
32	40.87%	10.37%
64	64.87%	16.80%

The optimal hyperparameter values are indicated in bold.

**Table 7:** Ablation study for Whisper-Small based on optimizers.

Optimizer	WER	CER
Adam	36.79%	9.92%
**RMSProp**	**28.67%**	**7.55%**

The optimal hyperparameter values are indicated in bold.

The observation of these ablation studies is as follows:

- **Learning rate:** the model performs well for the learning rate of 1e-5 with 40.87% WER because Whisper-Small is pre-trained on 680,000 hours of labeled audio; thus, it already possesses a vast phonetic and linguistic baseline. A high learning rate can overwrite these valuable pre-trained weights, leading to catastrophic forgetting.
- **Batch size:** the best performance is observed at a batch size of 16 (36.79% WER). Larger batch sizes (32 and 64) reduce stochasticity during optimization and may lead to suboptimal generalization. Given that Whisper-Small is pre-trained on large-scale noisy multilingual data, moderate batch sizes during fine-tuning help preserve robustness while adapting to the target Telugu speech domain.
- **Optimizers:** Adam applies momentum and bias correction, which can lead to updating the destructive parameters during the stage of tuning the model. For a low-resource language like Telugu, this practice could allow for excessive adaptation and moderate generalization on the test set. On the other hand, RMSProp relies on the current magnitudes of gradients to determine adaptive learning rates, which often results in established updates. It enables Whisper-Small to retain its pre-trained noise-robust representations while adapting to Telugu phonetics, resulting in lower WER of 28.67% and CER of 7.55% values.

### 4.3 Training configurations

The Wav2Vec XLSR-53 architecture was pre-trained on unlabeled data using specific configurations, including a mask_time_prob of 0.05 and attention_dropout and hidden_dropout values set to 0.00. A similar configuration was applied to the Whisper-Small model. Unlike Wav2Vec XLSR-53, which focuses on refining speech representations from large collections of unlabeled data, Whisper-Small is optimized for end-to-end learning. Table 8 highlights the key training parameters employed for both models. This comparison provides insight into the distinct fine-tuning strategies for each model.

**Table 8:** Optimal training parameters were used for the Wav2Vec XLSR-53 and Whisper-Small models.

Parameter	Wav2Vec XLSR-53	Whisper-small
Learning rate	3e-4	1e-5
Train batch size	16	16
Eval batch size	8	8
Seed	42	42
Gradient accumulation steps	2	2
Optimizer	RMSProp	RMSProp
LR scheduler type	Linear	Linear
LR scheduler warmup steps	400	400

## 5 Results and discussions

This section presents a comparative analysis of the performance of the Wav2Vec XLSR-53 and Whisper-Small models for Telugu speech recognition tasks.

Table 9 provides a comprehensive overview of the training loss, WER, and CER metrics for the Wav2Vec XLSR-53 model across 20800 training steps. The findings show that the model can optimize its internal representations over time, as seen by a constant decrease in training loss, WER, and CER. Specifically, the training loss exhibits a substantial decline from 1.1372 at step 1600 to 0.0792 at step 20800. This consistent decline in training loss values points to better model convergence and generalization. Furthermore, the WER demonstrates a consistent improvement throughout training, decreasing from an initial value of 60.96% at step 1600 to 27.69% at step 20,800. Similarly, the CER shows a gradual decline, improving from 15.41% at step 1600 to 6.76% at step 20,800. This decrease in WER and CER emphasizes how well the model can transcribe speech signal inputs, confirming that the effectiveness of the SSL paradigm works to improve speech representations and linguistic variances in the Telugu language.

**Table 9:** Training loss, WER, and CER across steps for the Wav2Vec XLSR-53 model.

Step	Training loss	WER	CER
1,600	1.1372	60.96%	15.41%
3,200	0.7883	41.89%	9.99%
4,800	0.5882	37.78%	8.97%
6,400	0.4618	35.85%	8.58%
8,000	0.3731	39.06%	8.88%
9,600	0.3084	35.08%	8.52%
11,200	0.2528	31.95%	7.75%
12,800	0.2081	32.27%	7.89%
14,400	0.1804	31.81%	7.70%
16,000	0.1457	30.03%	7.43%
17,600	0.1213	29.19%	7.26%
19,200	0.1000	28.23%	6.93%
20,800	0.0792	27.69%	6.76%

Table 10 illustrates the Whisper-Small model's performance metrics over 19,000 training steps, which shows a steady decrease in training loss, WER, and CER. As shown by the voice representations, the training loss decreases significantly, beginning at 0.1235 at step 1,000 and falling to 0.0000 at step 19,000. Furthermore, WER demonstrates a consistent improvement throughout training, decreasing from an initial value of 34.78% at step 1,000 to 28.70% at step 19,000. Similarly, the CER shows a gradual decline, improving from 8.82% at step 1000 to 7.49% at step 19,000. The Whisper-Small model learns meaningful speech representations, maximizing its capacity to transcribe spoken language with more precision, as seen by the gradual fall in training loss, WER, and CER. These findings highlight the model's stability during training, as evidenced by a gradual decrease in WER. The observed trends in training dynamics highlight the Whisper-Small model's efficiency in capturing linguistic and acoustic patterns.

**Table 10:** Training loss, WER, and CER across steps for the Whisper-Small model.

Step	Training loss	WER	CER
1,000	0.1235	34.78%	8.82%
2,000	0.0642	33.98%	8.88%
3,000	0.0337	33.65%	8.45%
4,000	0.0196	32.62%	8.41%
5,000	0.0126	31.67%	8.12%
6,000	0.0085	33.27%	8.41%
7,000	0.0063	30.34%	8.07%
8,000	0.0045	30.60%	7.97%
9,000	0.0033	30.15%	7.88%
10,000	0.0024	30.70%	8.00%
11,000	0.0019	29.17%	7.76%
12,000	0.0015	30.28%	7.92%
13,000	0.0011	29.69%	7.79%
14,000	0.0006	29.75%	7.84%
15,000	0.0005	29.56%	7.67%
16,000	0.0003	29.16%	7.61%
17,000	0.0002	28.91%	7.63%
18,000	0.0001	28.86%	7.56%
19,000	0.0000	28.70%	7.49%

**Figure 9:**
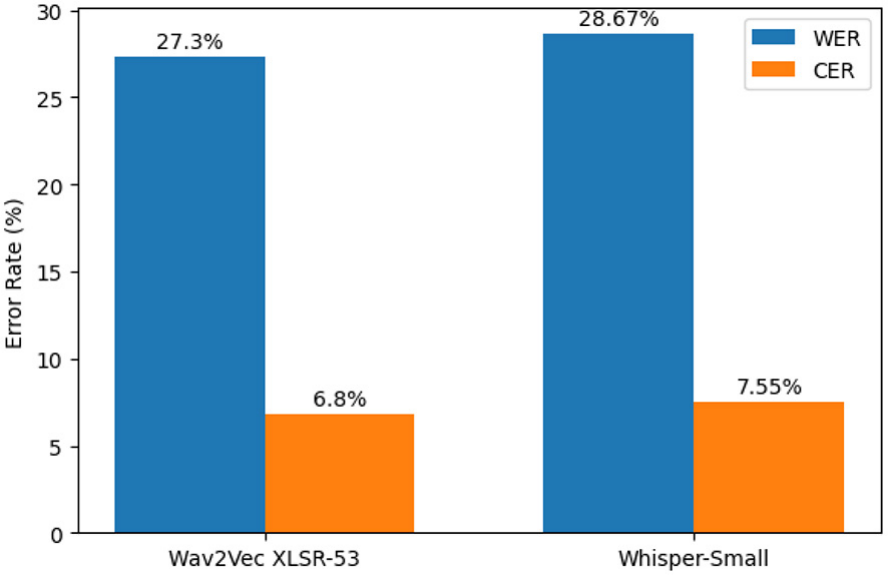
Comparison of WER and CER between the Wav2Vec XLSR-53 and the Whisper-Small models.

Figure 9 presents a comparison of the WER and CER metrics for the Wav2Vec XLSR-53 and Whisper-Small models, fine-tuned on the Telugu language. The results showed that Wav2Vec XLSR-53 achieves a WER of 27.03% and a CER of 6.8%, and Whisper-Small records a WER of 28.67% and a CER of 7.55%. This indicates that Wav2Vec XLSR-53 performs better because it is designed to learn fine-grained phonetic and acoustic representations from raw audio. Studies have shown that such representations transfer well to low-resource and single-language settings when the data is clean and carefully pre-processed. Fine-tuning benefits from reduced acoustic variability and stable speaker characteristics

### 5.1 Model evaluation observations

This section analyzes Telugu language performance through detailed qualitative evaluation and compound error analysis. The analysis highlights how errors accumulate across words, providing insights into model robustness and linguistics.

#### 5.1.1 Qualitative evaluation

**Figure 10:**
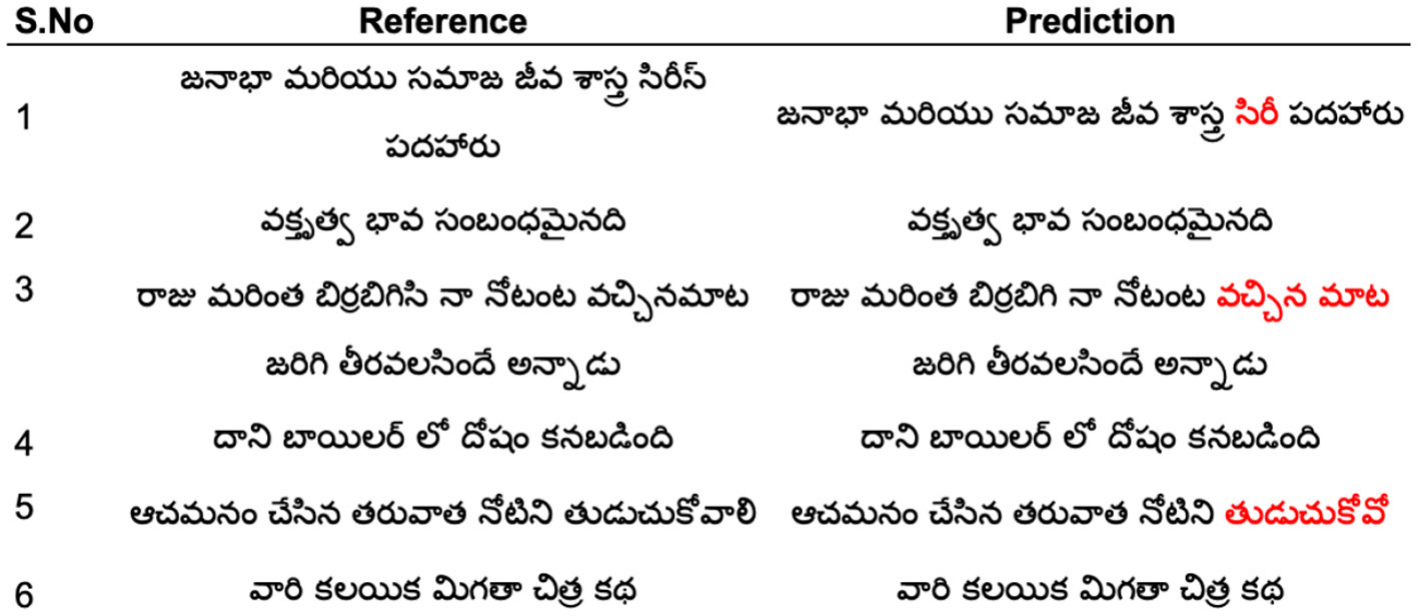
Selected reference vs. predicted transcriptions using the Wav2Vec XLSR-53 for Telugu (red-colored words indicate that the model mispredicts).

**Figure 11:**
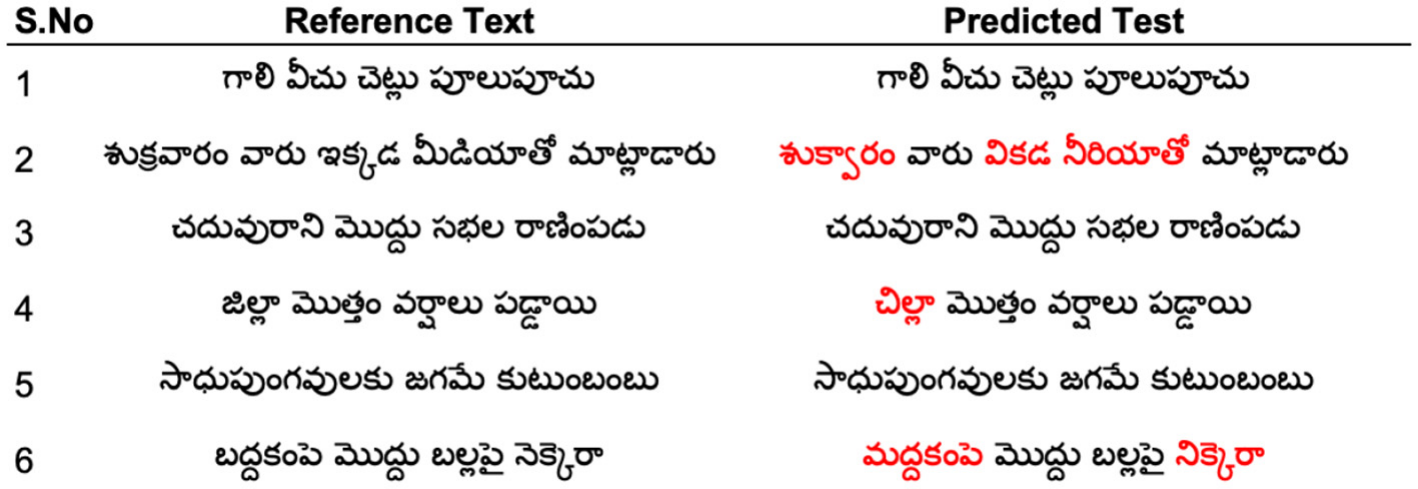
Selected reference vs. predicted transcriptions using the Whisper-Small for the Telugu (red-colored words indicate that the model mispredicts).

In our investigation of Telugu ASR, both models were compared and evaluated qualitatively on identical audio subsets drawn from the combined test data of six random audio clips. These examples allowed us to see how each model handled real-world utterances, including accent and vocabulary variations. Figure 10 presents the reference text and the predicted text of the fine-tuned Wav2Vec XLSR-53 model. It shows that Wav2Vec XLSR-53 also produced intelligible transcriptions for many sentences. In simpler segments, it often matched the ground truth word-for-word. However, it showed noticeable slips when faced with complex or compound expressions–some words were partially omitted or rearranged in a way that hinted at phonetic confusion. Interestingly, Wav2Vec XLSR-53 maintained consistent performance on repeated phrases within a single utterance, suggesting that it learned local patterns. Figure 11 presents the reference text and the predicted text of the fine-tuned Whisper-Small model. It illustrates that Whisper-Small generally did well with full phrases and slightly longer sentences. It appeared to handle continuous speech with fewer abrupt breaks, indicating robust internal language modeling. However, there were occasional errors where the model split compound Telugu words or substituted phonemes in a way that altered the meaning. Despite these issues, many predicted transcripts closely resembled the actual text.

#### 5.1.2 Error analysis

By examining the mispredictions highlighted in Figures 10, 11, we can identify three recurring challenges:

- **Compound word handling:** the Telugu language frequently merges multiple words into a single compound form. Both models sometimes struggled to correctly recognize these forms, leading to split or concatenated words.
- **Phonetic variations**: slight shifts in pronunciation or dialectal differences caused certain phonemes to be incorrectly recognized. This was visible in longer words with multiple syllables.
- **Contextual nuances:** words that rely on context (including idioms and culturally specific references) were occasionally mistranslated or omitted, reflecting the complexity of embedding such knowledge into an ASR model.

A key observation from our results is the large discrepancy between the CER and the WER for models. This phenomenon can be largely attributed to the agglutinative nature of the Telugu language. In agglutinative languages, morphemes (units of meaning) are often chained together to form long and complex words. A single character-level error, such as a misplaced vowel sign (*gunintam*) or an erroneous space, can cause the entire compound word to be marked as incorrect, thereby inflating the WER metric while only minimally affecting the CER. For the misrecognized sentences in Figures 10, 11, we have performed the error analysis in Table 11, which gives a qualitative analysis of common error patterns observed in the model's predictions. This analysis confirms that the high WER is not indicative of poor semantic understanding but is rather a byproduct of the model's difficulty with the morphological complexity of Telugu compound words.

**Table 11:** Error analysis of Whisper-Small and Wav2Vec XLSR-53 models.

Model used	Sen. No.	IPA (Ref.)	IPA (Pred.)	Translation	Error descrip.	Error type
Whisper	2	/shukra-vaaram vaaru ikkada meediya-to maatlaadaaru/	/shukva-ram vaaru vikkada neediya-to maatlaadaaru/	On Friday, they spoke to the media here	• shukra-vaaram → shukva-ram • ikkada → vikkada • meediya → neediya	• Consonant deletion • Consonant addition • Consonant substitution
	4	/jilla mottam paryaalu paddaayi/	/chilla mottam paryaalu paddaayi/	The entire district was affected	jilla → chilla	Consonant substitution
	6	/baddakampe medda ballapai nekkaraa/	/maddakampe medda ballapai nikkaraa/	Did you climb the table out of laziness	• baddakampe → maddakampe • nekkaraa → nikkaraa	• Consonant substitution • Vowel substitution
Wav2Vec	1	/janaabha mariyu samaaja jeeva shaastra series padahaaru/	/janaabha mariyu samaaja jeeva shaastra seeri padahaaru/	Population and society life science series sixteen	series → seeri	Word truncation
	3	/raaju marinta birrabigisi naa notanta vachinamaata jarigi tirvalasinde annadu/	/raaju marinta birrabigisi naa notanta vachina maata jarigi tirvalasinde annadu/	The minister stated firmly that the words he uttered must definitely happen	vachinamaata → vachina maata	Word split
	5	/aachamanam chesina tarvaata nootini tuduchukovaali/	/aachamanam chesina tarvaata nootini tuduchukovo/	After the ritual of sipping water, one should wipe their mouth	tuduchukovaali → tuduchukovo	Suffix substitution

#### 5.1.3 Performance noise augmentation

Table 12 presents the performance of the Whisper-Small and Wav2Vec XLSR-53 models evaluated on different noise-augmented datasets. The background noise was sourced from the ESC-50 dataset (Piczak, 2015). The experiments evaluate model robustness under noisy conditions using four augmented datasets. In Experiment 1, noise is added to 2.41 hours of Common Voice, where Whisper Small records 12.98% WER and 3.88% CER, while Wav2Vec XLSR 53 achieves a slightly lower WER of 11.65% and a clearly lower CER of 3.02%. In Experiment 2, noise is applied to 2.00 hours of Indic TTS, where Whisper Small performs strongly with 15.22% WER and 3.02% CER, whereas Wav2Vec XLSR 53 degrades significantly with 24.98% WER and 4.80% CER, showing lower robustness to synthetic noise conditions. In Experiment 3, noise is added to 3.13 hours of OpenSLR, where Whisper Small achieves 13.33% WER and 2.89% CER, outperforming Wav2Vec XLSR 53, which records 18.40% WER and 3.93% CER, indicating better alignment of Whisper with noise augumented data. In Experiment 4, all noise augmented datasets totaling 7.54 hours are combined, and Whisper Small maintains stable performance with 13.97% WER and 3.54% CER, while Wav2Vec XLSR 53 drops to 19.59% WER and 4.58% CER. Overall, Whisper Small consistently outperforms Wav2Vec XLSR 53 under noise due to its large-scale pretraining on 680k hours of labeled audio containing background noise, overlapping speech, reverberation, and recording variability, making noisy evaluation data closer to its pretraining distribution.

**Table 12:** Performance on the noise-augmented training data.

Experiment no.	Noise dataset	No of hours	Whisper-Small		Wav2Vec XLSR-53	
			WER	CER	WER	CER
1	Common Voice	2.41	12.98%	3.88%	11.65%	3.02%
2	IndicTTS	2.00	15.22%	3.02%	24.98%	4.80%
3	OpenSLR	3.13	13.33%	2.89%	18.40%	3.93%
4	Combined noise data	7.54	13.97%	3.54%	19.59%	4.58%

### 5.2 Comparison with the existing models

Table 13 presents a comparative analysis of our test results against previous efforts in Telugu ASR, emphasizing improvements. Earlier work Chowdary et al. (2024a) introduced a multilingual ASR model using Whisper, reporting a WER of 61.2% for Telugu. Yadavalli et al. (2022a) employed dialect-specific language models and achieved a WER of 34.3%, and a CER of 11.2%, while the Errgrams system proposed by Bhanuprasad and Svenson (2008), using an HTK-based large vocabulary recognizer on mismatched data, reported a WER of 40.4%. A transformer-based Wav2Vec XLSR-53 ASR model (Chadha et al., 2022) for building multilingual and code-switched ASR systems for Telugu achieved a WER of 29.39%. In a recent study, Jain and Bhowmick (2025) fine-tuned the Wav2Vec XLSR-53 and Whisper-Small models, achieving a WER of 28.67% and 30.04%, and a CER of 4.92% and 10.31%, respectively. In comparison, our fine-tuned Wav2Vec XLSR-53 and Whisper-Small models of test evaluation resulted in WERs of 27.3% and 28.67%, and corresponding CERs of 6.8% and 7.55% on the Telugu combined dataset. The proposed fine-tuned Wav2Vec XLSR-53 and Whisper-Small models demonstrate satisfactory performance over existing Telugu language models, achieving lower WER and CER.

**Table 13:** Comparison of ASR approaches for the Telugu language.

Reference	Model	WER	CER
Chowdary et al. (2024a)	Whisper	61.2%	NA
Yadavalli et al. (2022a)	Dialect-specific language models	34.3%	11.2%
Bhanuprasad and Svenson (2008)	HTK large vocabulary speech recognizer (mismatched)	40.4%	NA
Chadha et al. (2022)	Wav2Vec XLSR-53	29.39%	NA
Jain and Bhowmick (2025)	Wav2Vec XLSR-53	28.67%	4.92%
Jain and Bhowmick (2025)	Whisper-Small	30.04%	10.31%
Proposed Work	Wav2Vec XLSR-53	27.3%	6.8%
Proposed Work	Whisper-Small	28.67%	7.55%

### 5.3 Inference speed comparison

To evaluate latency and the real-time factor (RTF), audio samples of 10 seconds and 15 seconds were used with the Wav2Vec XLSR-53 and Whisper-Small models. Latency was measured by executing inference on the same audio inputs five times and reporting the average runtime. The RTF was calculated as the ratio of latency to audio duration. The results presented in Table 14 show that Wav2Vec XLSR-53 achieves superior inference speed compared to Whisper-Small.

**Table 14:** Model performance comparison based on audio duration.

Models	Audio duration	Latency	RTF
Whisper-Small	10	6.81	0.68
	15	6.51	0.43
Wav2Vec XLSR-53	10	2.73	0.27
	15	3.75	0.25

## 6 Conclusion

This study presented a comparative analysis of Wav2Vec XLSR-53 and Whisper-Small architectures for Telugu ASR. The proposed implementation marks a significant step toward developing robust ASR systems for the low-resource Telugu language. To enhance the robustness of the model, we integrated greater diversity in speaker demographics and linguistic content by utilizing four publicly available datasets: OpenSLR, Common Voice, IndicVoices, and IndicTTS. These datasets were combined, and the Wav2Vec XLSR-53 and Whisper-Small models were fine-tuned on the aggregated data. Experimental results indicate that the fine-tuned Wav2Vec XLSR-53 model achieves a WER of 27.3%, outperforming Whisper-Small, which attains a WER of 28.67%, and the CERs for Wav2Vec XLSR-53 and Whisper-Small are 6.8% and 7.55%, respectively. In the presence of noisy data, results from three out of the four augmented noise datasets indicate that Whisper-Small outperforms Wav2Vec XLSR-53. These findings underscore the impact of architectural design and hyperparameter optimization in enhancing model performance in low-resource settings. Also, the examination of suboptimal hyperparameter configurations provides critical insights into effective training strategies. In the future, a real-world application can be developed by collecting a larger Telugu speech corpus that covers all dialects of the language. Further work should evaluate model generalization on code-mixed and noisy speech and explore multilingual pretraining using Indic-centric corpora or dialect-specific fine-tuning strategies. The implementation of this work is available at the following GitHub repository: Code_for_the_project.

## Data Availability

The original contributions presented in the study are included in the article/supplementary material, further inquiries can be directed to the corresponding author.
